# Research on Regional Variations in Potato Price Fluctuations and Inter-Regional Transmission Mechanisms in China

**DOI:** 10.3390/foods14234135

**Published:** 2025-12-02

**Authors:** Hongwei Lu, Tingting Li, Ruoshi Hao, Zixuan Liu, Mingjie Gao, Junhong Chen

**Affiliations:** 1 Key Laboratory of Urban Agriculture (North China), Ministry of Agriculture and Rural Affairs, Institute of Data Science and Agricultural Economics, Beijing Academy of Agriculture and Forestry Sciences, Beijing 100097, China; luhongwei@baafs.net.cn; 2 Information Technology Research Center, National Engineering Research Center for Information Technology in Agricultural, Beijing Academy of Agriculture and Forestry Sciences, Beijing 100097, China; 3 Yunnan Provincial Research Institute of Plateau Specialty Agriculture, Yunnan Agricultural University, Kunming 650201, China; 4 State Key Laboratory of Efficient Utilization of Arid and Semi-Arid Arable Land in Northern China, Institute of Agricultural Resources and Regional Planning, Chinese Academy of Agricultural Sciences, Beijing 100081, China

**Keywords:** price volatility, potato, spatial variation, interregional transmission

## Abstract

Potatoes, possessing the characteristics of being suitable for food crop, vegetable, and fodder use, have become an important supplementary product for ensuring food security and vegetable supply. Their price fluctuations play a significant role in regulating production and consumption. Against the backdrop of establishing a unified national market, studying potato price fluctuations from a spatial perspective is crucial for scientifically and systematically understanding the patterns of China’s potato market. This study employs Ensemble Empirical Mode Decomposition, Spatial autocorrelation and Vector Autoregression models to analyse spatial variations and inter-regional transmission mechanisms in China’s potato price fluctuations, utilising wholesale market price data from January 2014 to December 2024 across diverse regions. Findings indicate distinct spatial patterns in potato price dynamics with significant inter-regional interactions. The Northern Crop Region exhibits predominantly short-term, high-frequency fluctuations, whereas the Central Crop Region, Southern Crop Region, and Southwestern Crop Region are characterized by long-term, low-frequency fluctuations. Potato prices in China exhibit significant spatial heterogeneity, and potato price fluctuations at both national and regional levels are primarily influenced negatively by those in other regions. The degree of interactive influence between potato prices across regions exhibits considerable variation, with the Central China crop region holding a certain degree of dominance in the national market. Based on these findings, policy recommendations are proposed, including strengthening tiered and regional monitoring and analysis of potato prices, standardizing inter-regional transmission pathways for potato prices, and guiding the formation of a complementary regional structure for potato production.

## 1. Introduction

Potatoes, with their high yield per unit area, rich nutritional value and strong environmental adaptability, constitute one of the world’s most important food sources [1,2]. In today’s world, marked by frequent international turmoil and increasing instability in global food industry chains and supply chains, the importance of potatoes in safeguarding food supply has become even more pronounced. China has now become the world’s largest producer and consumer of potatoes [3]. According to National Bureau of Statistics data, China’s potato cultivation area expanded from 2.88 million hectares to 4.56 million hectares between 1991 and 2022, representing an average annual growth rate of 1.49%. Concurrently, production rose from 6.081 million tonnes to 17.8826 million tonnes, achieving an average annual growth rate of 3.4%. Against the backdrop of increasingly stringent water and land resource constraints on China’s food crop production and diminishing growth potential for the three major staple crops, potatoes have emerged as the fourth staple crop with substantial yield expansion potential, capable of effectively stabilising the nation’s food crop supply capacity. Furthermore, China’s primary potato-producing regions exhibit significant spatial overlap with poverty-alleviation areas.

Significant fluctuations in agricultural commodity prices impact farmers’ income levels, reducing individual production capacity [4]. This hinders the transition of agricultural labour to manufacturing, undermines the stable economic development of developing countries [5], and poses a threat to national food security [6,7,8]. Moreover, price shocks may lead to reduced real income and increased costs across all sectors, rising unemployment rates, and heightened political instability [9,10]. Since the reform and opening-up, China has progressively deepened reforms in the price formation mechanisms for grain and key agricultural products, gradually developing a price support policy framework aligned with market economy principles. In its Action Plan for Deepening Price Mechanism Reform during the 14th Five-Year Plan period, the National Development and Reform Commission proposed establishing an agricultural production risk fund system to mitigate price volatility. Against this backdrop, multiple local governments, including Tengzhou City and Ulanqab City, have implemented targeted potato price insurance policies. These aim to enhance farmers’ resilience to market risks, promote increased production and income for farmers, and stabilise prices.

**Figure 1:**
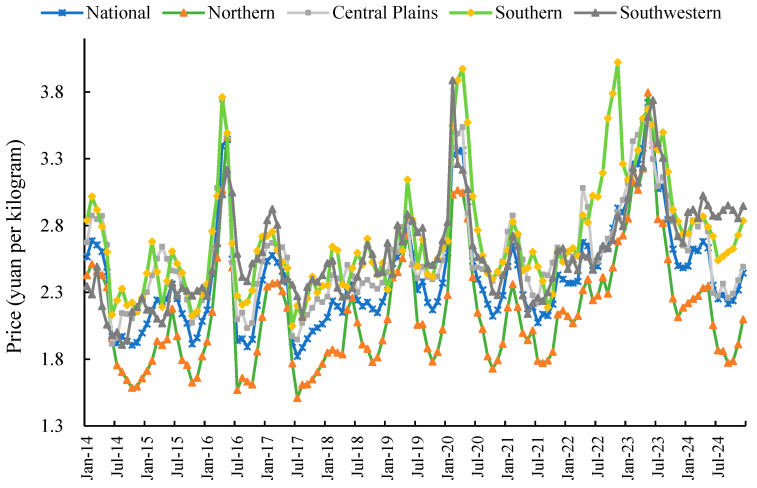
Monthly Price Trends for Potatoes by Region, 2014–2024.

From a temporal perspective, the relatively short growth cycle and pronounced seasonality of potatoes contribute to their price instability. Significant fluctuations in potato prices not only adversely affect farmers’ incomes but also constrain the stable and healthy development of the industry [11]. In recent years, China’s wholesale potato market prices have exhibited intense volatility. Over the past decade, monthly price differentials exceeding 1.5 yuan per kilogram occurred in 2016, 2020, and 2023 (Figure 1). Calculations indicate that the annual coefficient of variation for China’s monthly potato prices from 2014 to 2024 reached 11.13%. Potato production exhibits seasonal and dispersed characteristics, coupled with dependence on natural conditions. The predominance of small-scale farming places growers at a competitive disadvantage in the market. Spatial differentiation in the endowment structures of agricultural factors—such as land, labour, capital, and technology—across regions has led to spatial variations in agricultural output and quality, consequently resulting in spatial price disparities for agricultural products [12]. Analysis of monthly wholesale potato prices across China’s regional markets indicates two key patterns: on one hand, potato price fluctuations exhibit distinct characteristics in different regions; On the other hand, there exists a certain degree of consistency in the fluctuations of wholesale potato prices across different regions during most months, suggesting that prices in various areas may be interrelated.

This study aims to address the following scientific questions: What variations exist in the patterns of potato price fluctuations across different regions of China? How do prices propagate between regions from a spatial perspective? Only by thoroughly investigating these issues can we gain a more scientific and systematic understanding of the spatial patterns governing potato price fluctuations in China. To this end, this study employs an Ensemble Empirical Mode Decomposition (EEMD) model and Vector Autoregression (VAR) model to investigate the characteristics of potato price fluctuations across different regions of China from January 2014 to December 2024. It delves into the spatial transmission effects of potato price volatility. This research assists governments and relevant business entities in more accurately grasping the dynamics and evolving trends of the potato market across different regions. It provides reference for regulating potato market conduct and enabling relevant operators to optimise supply chain management and mitigate operational risks. This, in turn, contributes to stabilising potato supply-demand relationships and enhancing the degree of potato market integration.

Potential innovations of this paper: Firstly, it establishes a research framework for examining spatial variations and transmission mechanisms in potato price fluctuations, which may serve as a reference for similar studies on agricultural commodity price volatility and its spatial dynamics. Secondly, it pioneers a quantitative investigation of spatial disparities and inter-regional transmission in potato price fluctuations from a cultivation zone perspective, integrating research on the regional specificity of agricultural production with the spatial interconnectivity of markets, thereby expanding the traditional paradigm of agricultural commodity price studies. Thirdly, it pioneers the integration of the EEMD model and VAR model to examine agricultural price fluctuations, combining the EEMD model’s efficacy in analysing non-stationary data with the VAR model’s strengths in examining price transmission mechanisms.

## 2. Literature Review

Empirical research into agricultural price fluctuations can be traced back to the early 20th century. Within the analytical framework of traditional economic theory, time was regarded as a core variable, while the regularity and systematic nature of spatial dimensions were overlooked. Classical economists generally assumed complete mobility of production factors, positing that any regional disparities in prices, costs, and income would be swiftly eliminated through the full flow of factors. However, this assumption falls far short of the complexity of real economic activity. Empirical observations indicate that the ideal state of markets possessing perfect allocation mechanisms and complete factor mobility is difficult to achieve in practice [13]. The existence of spatial frictions in agricultural product circulation, stemming from information asymmetry, transport costs, and policy barriers, impedes the free flow of economic factors and the instantaneous adjustment of prices [14]. Consequently, agricultural product prices exhibit regional variations, and price levels across different regions may influence one another.

The methodological framework for studying agricultural price transmission mechanisms exhibits divergent modelling approaches. Early domestic research predominantly relied on linear analytical frameworks; however, empirical evidence indicates that agricultural price fluctuations exhibit pronounced non-linear characteristics [15,16], prompting academic re-evaluation of traditional linear assumptions. Regarding the drivers of price fluctuations, within the market supply-demand dimension, effective supply, consumer demand, inventory adjustments, and online sentiment serve as key drivers [17]. In terms of policy regulation, adjustments to agricultural and macroeconomic policies induce price volatility by altering market expectations [18]. Within the monetary environment, monetary rigidities and changes in money supply exhibit significant transmission effects on price fluctuations [19]. Regarding vertical transmission studies along industrial chains, a theoretical framework for price transmission between agricultural procurement and retail stages has been established [20], revealing short-term asymmetric and long-term balanced price transmission within the peanut oil industry chain [21]. Regarding methodological innovation, researchers proposed classifying price fluctuation frequencies and employing spectral analysis to test symmetry [22]. This confirmed asymmetric price transmission between production and retail ends in the US milk supply chain [23] and revealed market power-induced asymmetric price transmission in the fresh strawberry supply chain [24]. Price fluctuation spillover effects have also drawn attention. VAR and GARCH models analysed the US beef market, revealing the transmission mechanism of fluctuations between wholesale and retail segments [25]. GARCH models tested the correlation between agricultural inputs, primary agricultural products, and final retail prices [26]. EGARCH models confirmed the unidirectional transmission effect from the US feed market to the aquatic products market [27]. Chicago Mercantile Exchange futures prices serve as a core global benchmark for agricultural commodity pricing, exerting significant guiding effects on futures markets worldwide [28,29,30]. Research on the spatial integration of Brazil’s rice market confirmed overall equilibrium [31]. Policies that improve grain market efficiency in Russia should not only foster investments in transportation and trade infrastructure but also the development of market information services and commodity futures markets [32]. Analyzing the Indonesian rice market, we found that the contribution of the supply side was higher than that of the demand side [33].

Research on the spatial characteristics and transmission of agricultural commodity price fluctuations primarily covers three aspects: Firstly, spatial variations in price fluctuations. Most agricultural commodity prices exhibit regional characteristics. Pork prices across different provinces display spatial clustering, while fruit price volatility varies between northern and southern regions [34,35]. Chinese mutton prices indicate seasonal and spatial clustering features [36]. Secondly, the vertical transmission of agricultural product prices along supply chains. Studies examining cotton, maize, and other commodities from a supply chain perspective reveal asymmetric price transmission [37]. Other scholars have investigated price transmission mechanisms in rice and pork supply chains [38,39]. Some studies reveal no long-term equilibrium relationship between upstream and downstream prices in the maize supply chain [40], while the pig supply chain exhibits long-term asymmetry and short-term equilibrium [41]. Thirdly, spatial horizontal price transmission in agricultural products: international scholars have analysed global transmission through futures markets, finding that countries are influenced by Chicago Mercantile Exchange prices [28,29,30]. Within a single country, agricultural product prices across different regions exhibit long-term cointegration [42]. Spatial transmission is observed in Chinese citrus prices [43], while edible fungi market prices indicate correlation [44]. Domestic and international grain prices exhibit a long-term equilibrium relationship, with international grain prices exerting an asymmetric transmission effect on China [45,46].

Currently, considerable research has been conducted on potato price fluctuations in China, primarily focusing on temporal patterns and spatial variations. Firstly, regarding the characteristics and cyclical patterns of potato price fluctuations, research indicates that, between February 1996 and October 2007, wholesale market prices underwent approximately two complete cyclical phases, with an average cycle length of 70.5 months [47]. China’s potato price fluctuations exhibit significantly shorter cycles than the average for agricultural commodities, characterised by long-term stable increases coupled with short-term sharp volatility [48]. Additionally, scholars have analysed the annual, quarterly, and monthly fluctuations of wholesale potato prices alongside their cyclical characteristics [49], as well as the magnitude and frequency of price fluctuations [50]. Secondly, regional variations in potato price fluctuations and their causes have been examined [51], indicating a ‘higher in the south, lower in the north’ pattern across regions. Thirdly, some scholars have conducted predictive studies on potato prices [52,53]. Analysing potato price fluctuations from a spatial perspective represents a necessary endeavour to integrate spatial dimensions into the traditional theoretical framework for examining agricultural commodity price volatility. Traditional economic theories exhibit certain limitations in explaining these phenomena, prompting academia to explore integrating spatial dimensions into conventional economic frameworks. This aims to construct a more comprehensive economic analytical system better aligned with the real world [54].

Through analysis of relevant literature, existing research exhibits two primary shortcomings: firstly, studies on spatial variations in potato price fluctuations and their transmission mechanisms remain relatively underdeveloped. Consequently, potato price fluctuations may exhibit distinct spatial variations and correlations. Current research on potato price fluctuations has predominantly focused on temporal characteristics, with further investigation required into spatial variations and inter-regional transmission mechanisms. Secondly, existing methodologies for studying price fluctuations exhibit certain limitations. As an agricultural commodity, potato prices exhibit complex properties such as non-linearity and non-stationarity, characterised by overlapping fluctuation cycles [55]. Existing studies on potato price fluctuation characteristics and cyclical patterns predominantly employ traditional methods such as HP filtering, X-12 seasonal adjustment, and GARCH clusters [56,57]. These approaches fail to extract multi-timescale fluctuation patterns in potato prices. The Ensemble Empirical Modal Decomposition (EEMD) method, however, can decompose potato price fluctuations across different timescales based on the data’s inherent characteristics, comprehensively indicating the intrinsic volatility features of potato price data [58]. Introducing this method into the study of potato price fluctuations can further enhance the scientific rigour and effectiveness of research.

## 3. Data and Model

### 3.1. Data Source

The potato price data utilised in this study were sourced from the Ministry of Agriculture and Rural Affairs’ National Agricultural Product Wholesale Market Price Information System. The spatial scope encompasses approximately 180 designated wholesale markets nationwide, with data collected spanning 4015 days from 1 January 2014 to 31 December 2024, comprising daily price records from major wholesale markets. The data employed in this study thus possesses considerable breadth and authority. Daily price data from covered wholesale markets across each province underwent mean processing, ultimately yielding monthly potato price data for 28 provinces (excluding the Hong Kong S.A.R., Macao S.A.R., Taiwan Province, Qinghai Province, Xizang Autonomous Region, and Hainan Province).

China’s potato cultivation zoning is comprehensively delineated based on factors including the crop’s biological characteristics, cultivation systems, variety types, and geographical and climatic conditions [59]. This zoning scheme represents a scientific division and planning of potato cultivation areas, aiming to maximise regional advantages, enhance yield and quality, and promote sustainable industrial development. Presently, China’s potato zoning is primarily divided into four cultivation zones (Table 1). The following considerations underpin the study of price fluctuation differences and transmission patterns across distinct potato cultivation zones: Firstly, the first factor to consider is supply and demand dynamics. Variations in climatic conditions and cultivation practices across zones result in differing potato supply timings and volumes. For instance, the Northern Single-Crop Zone primarily harvests in autumn, whereas the Central Double-Crop Zone and Southern Double-Crop Zone feature both summer and winter harvest periods. These temporal supply variations influence market supply-demand dynamics, thereby affecting regional price levels. Secondly, cultivar selection and market preferences differ across zones. Dominant potato varieties cultivated in each region align with distinct market demands. For instance, the Northern Single-Crop Zone favours late-maturing or medium-late varieties, whereas the Central Double-Crop Zone and Southern Double-Crop Zone predominantly cultivate early-maturing types. Such varietal distinctions confer regional price advantages in meeting specific market requirements.

In accordance with the principles of representativeness and data availability and based on the potato zoning scheme, the Northern Single-Crop Zone (BF), Central Plains Double-Crop Zone (ZY), Southern Double-Crop Zone (NF), and Southwestern Mixed-Crop Zone (XN) collectively encompass the following provinces (excluding Hong Kong, Macao, and Taiwan), as detailed in Table 1. Among these, seven provinces—Liaoning, Hebei, Shanxi, Shaanxi, Hunan, and Hubei—span multiple cultivation zones. Their assigned research regions are determined based on their yield contribution to the respective zone exceeding half of the province’s total output. Monthly wholesale potato prices for each region are calculated as the arithmetic mean of data collected from designated Ministry of Agriculture and Rural Affairs markets within each cultivation zone.

### 3.2. Research Methodology

#### 3.2.1. Ensemble Empirical Mode Decomposition

In 1998, Huang and others, Chinese-American scientists, proposed a method of using EMD (Empirical Mode Decomposition) decomposition to deal with non-smooth and non-linear signals [60]. This method can decompose the fluctuation cycle law and long-term development trend embedded in the signal and obtain the IMF components of different time scales. Each component must satisfy two conditions at the same time: (i) the number of extreme points and the number of zero points are equal or differ by at most one; (ii) the upper and lower envelopes of the very large and very small values are zero at any one time. The specific steps are as follows:

(1) The upper and lower envelopes X_up_(t) and X_low_(t) are fitted using cubic spline functions.

(2) Calculate the mean m(t)

(1) m(t)= [X_up_(t) + X_low_(t)]/2

(3) Derive a new series h(t)

(2) h(t) = X(t) − m(t)

If h(t) satisfies the two previous conditions, h(t) is denoted as imf_1_(t). If h(t) does not satisfy, then replace X(t) with h(t) and carry out the previous process again until we find the imf_1_(t) that satisfies the previous conditions.

(4) Calculate

(3) r(t) = x(t) − imf_1_ (t)

Denote it as r_1_(t) and repeat the previous steps to get imf_2_(t), repeat n times to get.

(4) r_1_(t) − imf_2_(t) = r_2_(t) r_n−1_(t) − imf_n_(t) = r_n_(t)

(5) When imf_n_(t) or r_n_(t) is smaller than a predetermined value, or when r_n_(t) becomes a monotonic function, and the pattern function cannot be filtered out anymore, the above decomposition process is stopped.

Finally, the original signal X(t) is decomposed into n IMF components and a residual term *r_n_(t)*, namely

(5) $$X(t)=\sum_{n=1}^{N}{imf}_{i}\left(t\right)+r_{n}(t)$$

However, EMD decomposition has the disadvantage of modal confusion, which leads to the lack of thorough EMD decomposition, whereas the problem of modal confusion is overcome by the inclusion of applying white noise sequences [61], and we proposed the EEMD model, which makes it possible to decompose various types of signals efficiently and to obtain the fluctuation components and trend quantities with different time scales. The decomposition steps are as follows:

(1) Add the white noise sequence ω_i_(t) with Mean of 0 and SD of c to x(t), i.e.:

(6) *X*_i_ (t) = *x*(t) + *ω*_i_(t)

(2) After the EMD decomposition of x_i_(t), the IMF is integrally averaged to obtain the final IMF, i.e.:

(7) $$C_{j}(t)=\frac{1}{N}\sum_{n=1}^{N}C_{ij}\left(t\right)$$

#### 3.2.2. Vector Autoregressive Model

The EEMD model first serves as a diagnostic tool, revealing the multi-scale fluctuation characteristics inherent within each production area’s price sequence. This pivotal finding implies that inter-regional price transmission must be a complex process integrating multiple fluctuation patterns. Consequently, we subsequently employ a VAR model to capture the holistic dynamic transmission network across regions, derived from this original price sequence rich in multi-scale information. Vector autoregressive model, abbreviated as VAR model, is a commonly used econometric model, which was proposed in 1980 by Christopher Sims [62], who argued that before that, there were problems with model construction and selection that made the model distorted in reflecting the economic system, and lost the possibility of the model explaining the reality and making suggestions. The VAR model is a regression of all current variables in the model on a number of lagged variables of all variables. The VAR model has been widely used to study the dynamic feedback and transmission mechanisms of the variables in the economic system by means of a shock response analysis, which is used to estimate the dynamic relationships of the joint endogenous variables without any ex ante constraints.

The general expression is:

(8) $$Y_{t}\;=\;A_{1}Y_{t-1}+A_{2}Y_{t-2}+\cdots +A_{p}Y_{t-p}+B_{1}X_{t}+\cdots +B_{r}X_{t-r}+\varepsilon_{t}$$

where Y_t_ is a vector of m-dimensional endogenous variables of the national potato wholesale market price and X_t_ is a vector of d-dimensional exogenous variables of the potato wholesale market price in different regions, with endogenous variables and exogenous lags of order p and r, respectively; A_1_, A_2_, … A_p_ and B_1_, B_2_, … B_r_ are the matrices to be estimated; and ε_t_ is a random term. This study employs a Vector Autoregression (VAR) model to investigate the transmission patterns among monthly potato price variables across the Northern Single-Crop Region, Central China Double-Crop Region, Southern Double-Crop Region, and Southwestern Mixed-Crop Region from January 2014 to December 2024. It thereby identifies the dynamic interactive relationships between prices across these regions.

## 4. Results and Analysis

### 4.1. Analysis of Regional Variations in Potato Price Fluctuations in China

As shown in Figure 1 depicting the monthly price trends across national potato wholesale markets, from 2014 to 2024, potato wholesale prices exhibited frequent fluctuations, generally centred around RMB 2.5 per kilogram. Significant fluctuations occurred in 2016, 2020, and 2023, with the maximum price differential exceeding 1 yuan per kilogram. Analysis of regional monthly wholesale price variations across China from 2014 to 2024 (Figure 1) indicates pronounced spatial disparities in potato pricing and volatility. In terms of price levels, potato prices in China’s Northern First Crop Zone were generally lower than other regions during most months. Wholesale prices in the Central Plains Second Crop Zone were also typically lower than those in the Southern Second Crop Zone and the Southwest Mixed Cropping Zone. Meanwhile, wholesale prices in the Southern Second Crop Zone and the Southwest Mixed Cropping Zone exhibited an inverse relationship, with one rising as the other fell. Regarding price volatility, while certain similarities exist across regional wholesale markets, overall price fluctuation patterns differ significantly. The Southwest mixed-cropping region and Central Plains second-crop region exhibit relatively minor price fluctuations, whereas the Northern primary-crop region and Southern second-crop region experience more pronounced and frequent price swings. The primary reasons may be that the Northern Single-Crop Region features large-scale cultivation and higher mechanisation levels, resulting in relatively lower production costs. Additionally, potato harvests in this region are concentrated, leading to substantial short-term market supply. Conversely, potato cultivation in the Southern Double-Crop Region and the Southwest Mixed-Crop Region faces greater constraints from natural conditions, incurring higher production costs. Furthermore, significant differences in natural conditions between these two regions cause variations in potato growth cycles and harvest timing.

From the perspective of cultivation zones, examining regional price fluctuation patterns holds practical significance. As illustrated in Figure 1, overall similarities exist in wholesale market price fluctuations across China’s potato-growing regions. However, at the short-term scale, distinct characteristics emerge in regional potato wholesale market price movements. Potato price fluctuations in the Northern Single-Crop Zone and Central Plains Double-Crop Zone exhibit smoother patterns, characterised by longer cycles and smaller amplitudes. Conversely, the Southern Double-Crop Zone and Southwestern Mixed-Crop Zone display more pronounced price volatility, featuring shorter cycles, higher frequency, and more significant amplitude variations. Across different regions, influenced by long-standing consumption habits and natural geographical conditions, the production and market release cycles for potatoes within each region are relatively similar. However, potato prices exhibit distinct characteristics in their fluctuations between different regions. While the time-series variation diagram provides an initial understanding of the spatial differences in potato price fluctuations across China, more in-depth and rational judgements require further quantitative analysis.

To further analyse the spatial variation patterns of high- and low-frequency fluctuations in China’s potato prices, an integrated empirical mode decomposition method was employed to decompose wholesale market price data from different regions across China between January 2014 and December 2024. Each original time series yielded five characteristic modal components (IMF components). To examine the patterns of short- and long-term fluctuations across regions, the Fine-to-coarse reconstruction method [63] was applied to the IMF sub-series derived from the potato price decomposition. This yielded IMF components representing both high-frequency short-term and low-frequency long-term potato price fluctuations for each region.

#### 4.1.1. Spatial Variation in High-Frequency Short-Term Fluctuations

**Figure 2:**
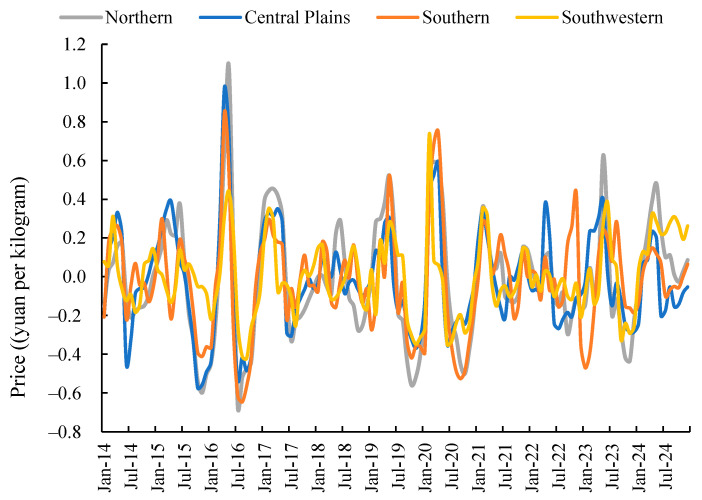
High-Frequency Components of Monthly Potato Price Fluctuations by Region, 2014–2024.

The coefficient of variation for high-frequency price fluctuations in potatoes exhibits spatial variation (Figure 2), with regional coefficients displaying an approximate four-year fluctuation cycle. Across the entire study period, the Northern Single-Crop Region exhibited the highest coefficient of variation, followed by the Central Double-Crop Region. The Southern Double-Crop Region also recorded a coefficient approaching 30%, while the Southwest Mixed-Crop Region registered 20.52%. This indicates that short-term, high-frequency fluctuations in wholesale potato prices are most pronounced in the Northern Single-Crop Region, followed by the Southern and Central Double-Crop Regions. Prices in the Southwest Mixed-Crop Region indicate greater stability compared to other areas. This may stem from the Northern Primary Production Zone’s relatively homogeneous potato cultivation structure, exhibiting pronounced seasonal supply patterns with concentrated harvest periods, leading to more pronounced market price fluctuations. Conversely, the Southwest Mixed Production Zone features a more diversified potato cultivation structure, with market supply lacking distinct seasonal characteristics, thereby maintaining greater price stability. From 2014 to 2024, the monthly price coefficient of variation for potato wholesale markets across all four regions exhibited a cyclical pattern of ‘increase-decrease-increase-decrease’ approximately every four years. The monthly price coefficient of variation for wholesale potatoes in the Northern Single-Crop Zone and Central China Double-Crop Zone peaked in 2016, while the Southern Double-Crop Zone and Southwest Mixed-Crop Zone recorded their highest coefficients in 2020. This cyclical pattern in the coefficient of variation may be influenced by agricultural production, climatic phenomena (such as El Niño), and periodic shifts in market demand.

Potato price fluctuation cycles exhibit marked regional variations. Analysis of variance contribution rates from high-frequency fluctuations across different regions (Table 2) indicates that short-term high-frequency fluctuations dominate in the Central China Second Cropping Zone. Monthly wholesale market price cycles for potatoes in the Northern First Cropping Zone, Central China Second Cropping Zone, Southern Second Cropping Zone, and Southwestern Mixed Cropping Zone exhibit fluctuation periods of 9.43 months, 8 months, 5.74 months, and 5.5 months respectively. The variance contribution rates indicate that high-frequency components account for 37.76% of total price fluctuations in the Northern First Crop Region’s potato wholesale market. With a correlation coefficient of 0.603 against the original price series, this region exhibits significant correlation between wholesale prices and original series fluctuations, though short-term high-frequency movements do not hold dominant influence. In the Central Second Cropping Region, the variance contribution rate of the high-frequency component exceeded 45.1%, with a correlation coefficient of 0.68, indicating its dominant influence on overall fluctuations and significant correlation. The variance contribution rate of the high-frequency component in the Southern Second Cropping Zone was 32.1%, while that of the high-frequency short-term fluctuation component in the Southwestern Mixed Cropping Zone was only 26.2%. This indicates that short-term high-frequency fluctuations do not play a dominant role in the Southern Second Cropping Zone and the Southwestern Mixed Cropping Zone. Overall, the variance contribution of high-frequency short-term fluctuations to the original series was below 40% in all three regions. This indicates that long-term low-frequency fluctuations exert significant influence on price volatility within each area, necessitating further investigation into the overall characteristics of these long-term low-frequency components across regions.

**Table 2:** Characteristics of High-Frequency Short-Term Fluctuations by Region and Their Correlation Coefficients with the Original Series.

Project	Year	Northern	Central Plains	Southern	Southwestern
Variation coefficients /%	2014	13.578	22.502	15.751	13.344
	2015	35.785	36.164	24.437	7.361
	2016	54.036	49.597	50.378	28.813
	2017	27.491	23.049	14.788	16.050
	2018	17.174	6.983	11.458	11.559
	2019	39.116	23.865	30.510	22.579
	2020	40.753	32.017	48.317	31.720
	2021	13.874	14.346	13.538	14.967
	2022	12.877	19.872	19.360	6.838
	2023	30.654	22.752	24.755	22.542
	2024	13.833	15.598	8.663	6.758
	Total	30.494	26.691	26.766	19.580
Average cycle (months)		9.429	8.000	5.739	5. 500
Variance contribution rate (%)		37.759	45.098	32.093	26.174
Pearson correlation coefficient		0.603 **	0.676 **	0.585 **	0.497 **

Notes: ** indicates that the correlation is significant at the 0.01 level.

**Figure 3:**
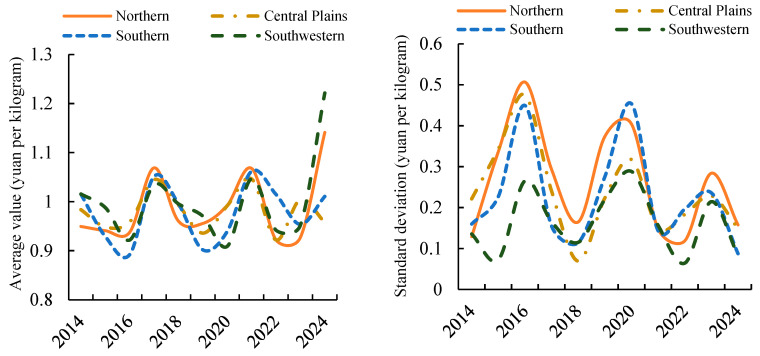
Mean and Standard Deviation of High-Frequency Short-Term Fluctuations by Sub-Region.

The mean and standard deviation of short-term potato price fluctuations across different regions (Figure 3) indicate certain similarities in their patterns of change. Regarding mean variations, the short-term price fluctuations in each region generally follow a four-year cycle. In recent years, the amplitude of mean fluctuations in potato prices across regions has shown an increasing trend. Among these, the Southwest Mixed Cropping Zone and Central Plains Second Cropping Zone exhibit relatively minor changes in the mean of short-term fluctuations, while the Northern First Cropping Zone indicates more pronounced variations in potato price fluctuations. Regarding standard deviation changes, from 2014 to 2024, the standard deviation of short-term potato price fluctuations across the four regions showed a narrowing trend. Among these regions, the Northern Single-Crop Region exhibited a larger standard deviation, with its variation also being greater than that of the other regions. The Southwest Mixed-Crop Region had a smaller standard deviation, and its numerical changes were relatively stable.

#### 4.1.2. Spatial Variation in Low-Frequency Long-Term Fluctuations

**Figure 4:**
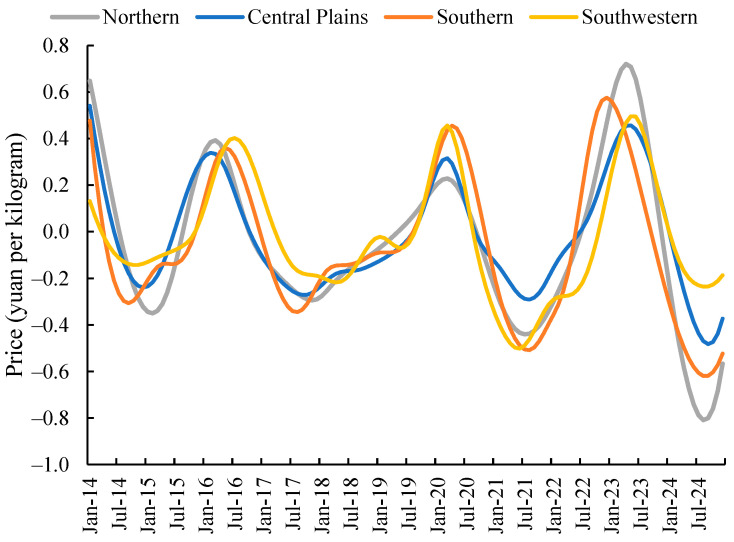
Low-Frequency Components of Monthly Potato Price Fluctuations Nationwide and by Sub-Region, 2014–2024.

As shown in Figure 4, at the low-frequency long-term scale, the Northern First Crop Region exhibits the highest monthly price coefficient of variation in wholesale markets, while the Central Plains Second Crop Region indicates the lowest. The coefficients of variation for low-frequency fluctuation components across regions follow an approximate four-year cyclical pattern. Analysis of the monthly price coefficient of variation for potato wholesale markets from 2014 to 2024 indicates that, over the long term, the Northern Single-Crop Region exhibits the highest monthly price coefficient of variation, followed by the Southern Double-Crop Region. The coefficients for the Southwestern Mixed-Crop Region and Central Plains Double-Crop Region stand at 40.94% and 36.6% respectively. This indicates that, over extended periods, potato wholesale market prices in the Central Plains second-crop region and Southwest mixed-crop region exhibit greater stability than the other two areas. Regarding low-frequency fluctuations, the Northern Single-Crop Region exhibited the highest coefficient of variation in 2024 at 47.1%. The Central Double-Crop Region recorded its peak coefficient of 24.6% in 2014. Both the Southern Double-Crop Region and the Southwestern Mixed-Crop Region reached their maximum coefficients in 2022, at 262.5% and 32.7% respectively.

**Figure 5:**
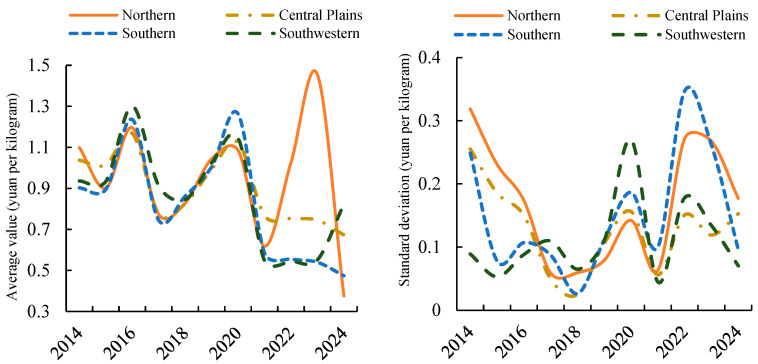
Mean and Standard Deviation of Sub-regional Low-frequency Long-term Fluctuations.

From the perspective of low-frequency long-term fluctuations, potato prices across regions exhibit pronounced long-cycle oscillations. The primary influence on raw price variations stems from low-frequency long-term fluctuations in the Northern Single-Crop Zone, Southwestern Mixed-Crop Zone, and Southern Double-Crop Zone (Table 3). Analysis of the periodic patterns of low-frequency fluctuations across regions indicates that the monthly wholesale market prices for potatoes in the Northern Single-Crop Region, Central Double-Crop Region, Southern Double-Crop Region, and Southwest Mixed-Crop Region exhibit long-term fluctuation cycles of 29.33 months, 29.33 months, 33 months, and 26.4 months respectively. The Southern Double-Crop Region exhibits the longest fluctuation cycle, followed by the Northern Single-Crop Region and Central Double-Crop Region, with the Southwest Mixed-Crop Region exhibiting the shortest cycle. Further analysis of variance contribution indicated that the low-frequency components in the Northern Single-Crop Region, Southern Double-Crop Region, and Southwestern Mixed-Crop Region accounted for 47.3%, 43.7%, and 40.9% of the variance in the original series, respectively. Pearson correlation coefficients exceeded 0.55 for all regions, indicating significant long-term fluctuations in the Northern Single-Crop Region, Southwestern Mixed-Crop Region, and Southern Double-Crop Region, with these long-term fluctuations dominating the variations in the original series. Examining the changes in mean and standard deviation of long-term potato price fluctuations across the four regions (Figure 5), the mean price volatility shows an increasing trend across all areas, with the Northern Region 1 exhibiting the most pronounced variation. The changes in standard deviation of long-term potato price fluctuations across the four regions, however, present a more complex pattern.

**Table 3:** Characteristics of Low-Frequency Component Fluctuations by Sub-Region and Their Correlation Coefficients with the Original Series.

Project	Year	Northern	Central Plains	Southern	Southwestern
Variation coefficients /%	2014	28.981	24.597	27.556	9.509
	2015	25.608	18.321	8.726	5.683
	2016	14.525	12.845	8.658	6.790
	2017	7.610	6.389	11.723	12.016
	2018	7.167	3.047	3.065	7.692
	2019	7.583	10.667	11.102	10.818
	2020	13.115	13.910	14.792	23.626
	2021	10.233	7.332	17.138	8.177
	2022	26.511	20.014	62.480	32.689
	2023	18.394	15.971	48.420	24.308
	2024	47.097	22.687	20.173	8.534
	Total	35.517	24.156	32.688	25.351
Average cycle (months)		29.333	29.333	33.000	26.400
Variance contribution rate (%)		47.303	36.601	43.721	40.940
Pearson correlation coefficient		0.597 **	0.588 **	0.584 **	0.563 **

Notes: ** indicates that the correlation is significant at the 0.01 level.

### 4.2. Analysis of the Spatial Price Transmission Mechanism for Potatoes in China

#### 4.2.1. Analysis of Spatial Correlation Across Provincial Regions

The First Law of Geography posits that all phenomena between regions exhibit inherent connections. Economic performance within a region and its neighbouring areas indicates strong autocorrelation, and the distribution of agricultural commodity prices may likewise display spatial correlation. The intensity of this correlation is closely linked to the degree of market integration [64]. Historically, scholars have predominantly focused on temporal patterns of agricultural price fluctuations, with limited attention paid to spatial distribution disparities. Research examining interrelationships between agricultural prices across different spatial units remains scarce [65]. Drawing upon economic geography theory, potato prices in neighbouring regions may exhibit a degree of correlation, forming patterns of spatial clustering and dispersion in potato prices. Spatial autocorrelation analysis enables the quantitative examination of potato price spatial distribution and its evolutionary patterns.

##### Global Autocorrelation Analysis

**Figure 6:**
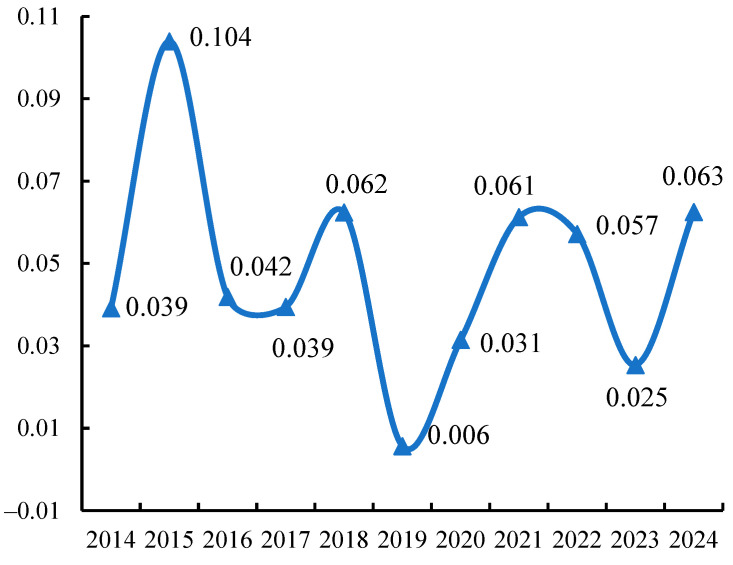
Changes in Moran’s I index of monthly potato prices in provincial areas during 2014–2024.

As indicated by the preceding analysis, potato prices in China exhibit spatial heterogeneity. We further employ exploratory spatial data analysis methods to examine the interannual variations in spatial correlation and spatial clustering characteristics of provincial potato prices. First, utilising GeoDa 1.20 software, a standard normalised statistic was constructed via repeated random permutations. A spatial weighting matrix was established using the Rook criterion for spatial adjacency matrices [66], enabling calculation of the global Moran’s I index for monthly potato prices. As illustrated in Figure 6, China’s provincial monthly potato price Moran’s I index indicates a significant positive spatial correlation. From 2014 to 2024, the index exhibited a cyclical pattern of initial increase, subsequent decline, and renewed rise, with spatial price correlations generally fluctuating within a 3- to 4-year cycle. Notably, the spatial correlation index peaked in 2016 and reached its lowest point in 2019. This indicates pronounced spatial heterogeneity in potato price fluctuations, with spatial disparities exhibiting cyclical patterns of expansion and contraction. In recent years, spatial heterogeneity in China’s potato market prices has been intensifying, reflecting markedly pronounced spatial variations within the market.

**Figure 7:**
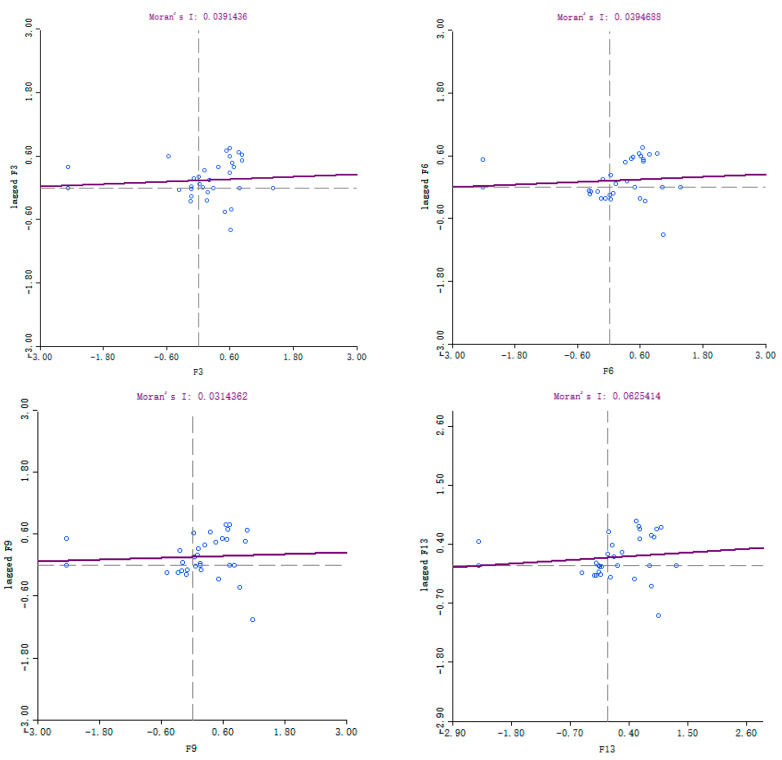
Moran Scatter chart of monthly potato prices in China’s provinces, 2014–2024.

Global autocorrelation analysis merely examines the spatial correlation characteristics of China’s monthly potato prices since 2014 at a macro-level, yet it fails to effectively indicate the local features of spatial clustering. Local spatial autocorrelation analysis effectively addresses the shortcomings of traditional methods by conducting an in-depth analysis of regional spatial clustering characteristics through Moran’s scatter plots and LISA clustering maps. Utilising the GeoDa software platform, this study constructed Local Moran’s I scatter plots (Figure 7) for national monthly potato prices in 2014, 2017, 2020, and 2024. Provincial monthly potato prices served as the horizontal axis variable, while the corresponding spatial lag vector (W) of prices formed the vertical axis variable.

The scatter plots delineate four spatial clustering types across four quadrants: low-low clustering (L-L), low-high clustering (L-H), high-high clustering (H-H), and high-low clustering (H-L). Observers distributed in the first and third quadrants exhibit spatial positive correlation, indicating similarity in their attribute characteristics across spatial distribution. Conversely, the second and fourth quadrants reflect spatial negative correlation, signifying significant divergence in attribute characteristics across spatial distribution. Scatter plot analysis indicates a prominent proportion of provinces located in the ‘H-H’ quadrant, reflecting a significant high-value clustering effect in provincial-level monthly potato prices. Spatial positive correlation was particularly pronounced in typical years. As illustrated, in 2014, 2017, 2020, and 2024, provinces classified as ‘H-H’ and ‘L-L’ types accounted for 60%, 63%, 67%, and 60% of the national sample total respectively. This indicates that inter-provincial monthly price patterns in China predominantly exhibit ‘H-H’ and ‘L-L’ clustering, with a high degree of spatial positive correlation in potato prices.

##### Local Autocorrelation Analysis

**Figure 8:**
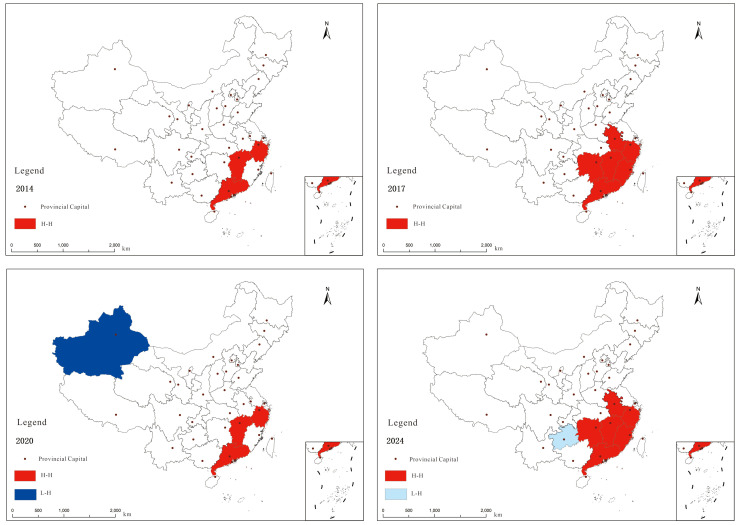
LISA clustering of monthly potato prices by provinces in China from 2014 to 2024.

LISA clustering maps can be employed to analyse the clustering patterns of regions with similar attributes within different spatial units. To systematically examine the local spatial clustering characteristics of monthly potato prices across provinces, this study calculated LISA indices for provincial monthly potato prices in 2014, 2017, 2020, and 2024 using the GeoDa software platform. Statistically significant LISA clustering types were identified through Z-statistic testing (*p* < 0.05). Analysis results (see Figure 8) indicate that most provinces’ monthly potato prices exhibit no significant local spatial clustering characteristics. Regions with strong spatial correlation remain limited in number, and provinces displaying positive spatial association show relatively stable distribution, primarily concentrated in the East China and South China economic zones. Regarding spatial clustering types, the ‘H-H’ pattern dominated in 2014 and 2017, while 2020 and 2024 were characterised by ‘H-H’ and ‘L-H’ and ‘H-L’ clusters. This indicates that, from 2014 to 2024, China’s monthly potato prices predominantly exhibited high-value clustering, meaning regions with higher monthly potato prices showed a geographically concentrated distribution pattern. In 2020 and 2024, provinces with low-value prices appeared surrounding those with high-value provinces in terms of coefficient of variation. Provinces consistently classified in the ‘H-H’ cluster from 2014 to 2020 included Guangdong, Jiangxi, and Zhejiang. Additionally, in 2017 and 2024, the ‘H-H’ cluster also included Hunan, Anhui, and Fujian provinces. This indicates that these provinces generally exhibited higher monthly potato prices across most years and maintained relatively close price correlations with neighbouring provinces, exerting a certain degree of radiating influence and driving effect on surrounding regions.

#### 4.2.2. Interregional Transmission Analysis

Markets serve as venues for transactions, while market systems constitute the regulatory framework governing such exchanges. The efficacy of market mechanisms in achieving resource utilisation and allocation efficiency depends not only on the transaction systems and underlying infrastructure conditions but also on the commodities and resources being traded, as well as the methods employed. As China’s economy enters a transitional phase and marketisation continues to advance, the flow of production factors and agricultural products between provinces accelerates. This may lead to interdependent and mutually influential price patterns among agricultural products across major production regions. Building upon an analysis of spatial variations in potato price fluctuations, this study further employs a VAR model to investigate the transmission mechanism of wholesale potato market prices across different regions.

##### Stability Test

The ADF test was employed to analyse the stationarity of different variable time series data, thereby assessing whether each time series was stationary. As shown in Table 4, the t-statistics for all variables fell below the critical values at various significance levels, indicating that the original sequences of each variable exhibited stationarity.

##### Determination of Lag Order

Establishing the lag order for the VAR model is a critical step in ensuring its validity. Should the residuals exhibit autocorrelation, setting the lag order too low may result in inconsistent parameter estimates; conversely, setting it too high reduces the model’s degrees of freedom and compromises parameter validity. The lag order was determined using AIC, LR, and FPE criteria (Table 5). Results indicate that the optimal lag order for the model is 2.

##### Stability Test of Equations

**Figure 9:**
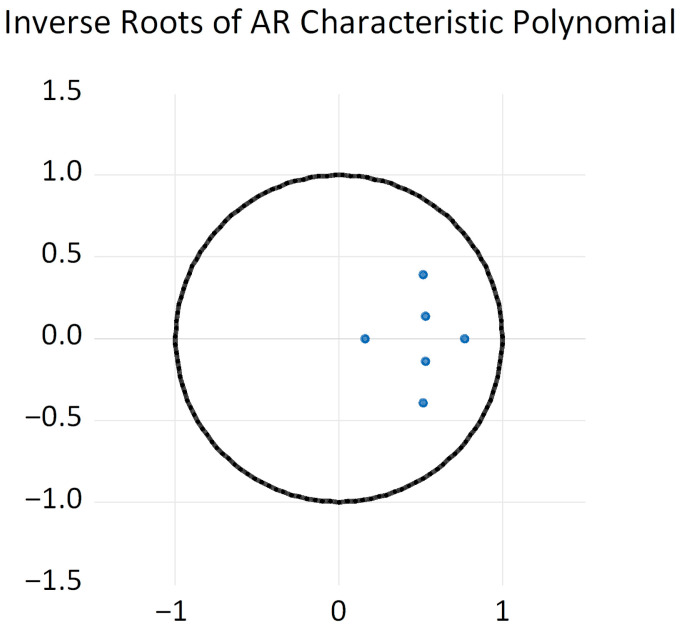
Equation Stability Test.

After determining the lag order of the VAR model, the AR root plot method was employed to further examine the stability of the equations (Figure 9). It was found that all characteristic roots lie within the unit circle, meaning the reciprocals of the AR coefficients are less than 1. This confirms the stability of the constructed VAR model. Subsequently, impulse response analysis and variance decomposition analysis were conducted.

##### Impulse Response Analysis

**Figure 10:**
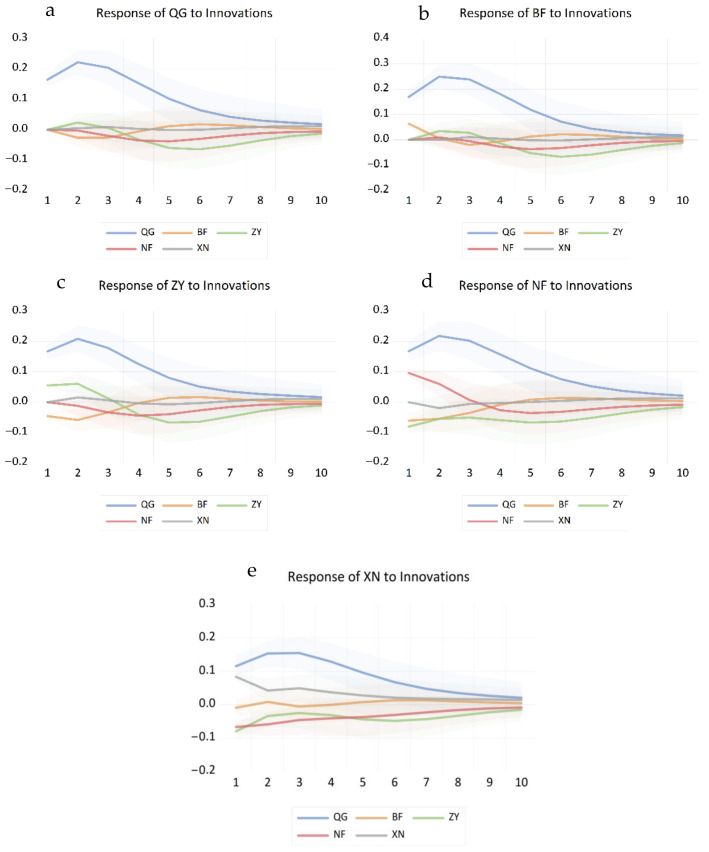
Impulse Response Diagram of Potato Price Fluctuations Across Different Regions. Notes: QG represents the National, BF represents the Northern, ZY represents the Central Plains, NF represents the Southern, and XN represents the Southwest.

Impulse response analysis provides a more intuitive representation of the dynamic interactions between various variables. An impulse response analysis of national and regional potato price data is presented in Figure 10, where the horizontal axis denotes the lag period of the impulse response, and the vertical axis indicates the magnitude of change in potato prices following an impulse. The solid line represents the impulse response function, illustrating the responses generated by mutual price shocks across different regions.

As shown in Figure 10a, during the first three months of national potato price fluctuations, the Central Plains second-crop region, the Southwest mixed-cropping region, and the national price exhibit synchronous movements. The national potato price indicates the strongest response to its own shocks. Within the initial three months, the Central Plains second-crop region and the Southwest mixed-cropping region exert a positive impact on the national price, while other regions exhibit a negative response to this positive shock. From an economic perspective, when national prices rise due to a shock, consumers in some regions may initially switch to potatoes from those areas due to market substitution effects. However, if supply in these regions fails to adjust promptly, cross-regional arbitrage activities rapidly intervene—arbitrageurs transport products from low-price areas to high-price markets. This may conversely lead to a relative tightening of supply in the original region followed by a price correction, resulting in a negative response to a positive shock. As shown in Figure 10b, potato price changes in the Northern Primary Production Zone are initially influenced in the same direction by national and Central Secondary Production Zone price movements. This zone exhibits the strongest response to national potato price shocks. From Period 3 to Period 6, the Northern Primary Production Zone primarily experiences negative responses to positive shocks originating from itself, the Central Secondary Production Zone, and the Southern Secondary Production Zone. This phenomenon can be explained by inter-regional arbitrage behaviour: when national or Central Plains second-crop region prices rise, the Northern first-crop region, as a major production area, initially follows suit with price increases. However, as arbitrage capital inflows drive up local procurement prices, the subsequent concentrated market release of produce or supply supplementation from other regions reverses the market supply-demand dynamics. This leads to a negative adjustment in response to the earlier positive shock. As shown in Figure 10c, potato price changes in the Central Second-Crop Region are initially influenced by both national and regional price movements in the same direction. Subsequently, they exhibit a negative response to positive shocks originating from the Northern First-Crop Region and Southern Second-Crop Region. The Central Region’s secondary crop area exhibited the strongest response to national potato price shocks. During the initial four periods, it primarily experienced negative reactions to positive shocks originating from the Northern primary crop area and Southern secondary crop area. This stems from the dynamic evolution of market substitution effects: when prices rise in the Central Region’s second-crop area, consumers may switch to substitute products from northern or southern production areas, driving short-term price increases in these substitute regions. However, once supply expands in these substitute areas, market competition exerts countervailing pressure on prices in the Central Region’s second-crop area, manifesting as a negative response. As shown in Figure 10d, potato price changes in the Southern Second-Crop Zone were initially influenced by both national and regional price movements in the same direction while also exhibiting a negative response to positive shocks from the Northern First-Crop Zone and the Southwestern Mixed-Crop Zone. Southern secondary crop region potato prices exhibit the highest responsiveness to national and regional price shocks. During the initial three periods, this region primarily experienced negative responses to positive shocks from the other three zones, with the impact gradually diminishing after the eighth month. From a cross-regional arbitrage perspective, during the initial phase of price increases in the Southern Second Cropping Region, potatoes from producing areas such as the North and Southwest enter this market via cross-regional transport. This increases local supply, thereby suppressing further price rises and forming a negative response to positive shocks. As market supply and demand gradually balance, arbitrage opportunities diminish, and the impact effect naturally weakens. As shown in Figure 10e, price changes in the Southwest Mixed-Crop Zone were initially influenced by both national and local price movements in the same direction while also experiencing a negative response to positive shocks originating from other regions. During the first five periods, the Southwest Mixed-Crop Zone primarily exhibited a negative response to positive shocks from the other three regions, with this impact gradually diminishing after the eighth month. This closely relates to market substitution effects: when prices fluctuate in the Southwest Mixed Cropping Region, potatoes from neighbouring and northern production areas become substitute options due to differences in transport costs or market entry cycles. As the market share of substitute products expands, prices in the Southwest Mixed Cropping Region experience a countervailing effect. However, this substitution effect gradually diminishes as seasonal changes occur or market demand stabilises.

The above indicates that, following a price shock, potato prices across all regions typically reach their peak response in the second period, with the impact effect diminishing after approximately eight months. Prices across regions exhibit mutual influence, though the direction and magnitude of this influence vary by time and location. Regions may initially experience positive shocks from others, later shifting to negative responses to such positive shocks. This dynamic evolution fundamentally stems from the combined effects of market substitution and cross-regional arbitrage: price increases in one region drive short-term price rises elsewhere through substitution demand. However, increased supply from cross-regional arbitrage subsequently pulls prices back towards equilibrium levels, generating negative responses. The findings indicate a high degree of integration within the potato market, with significant inter-regional price linkage effects. This implies that price changes in one region may rapidly propagate to others through market substitution and cross-regional arbitrage, thereby influencing overall market price levels.

##### Variance Decomposition

**Figure 11:**
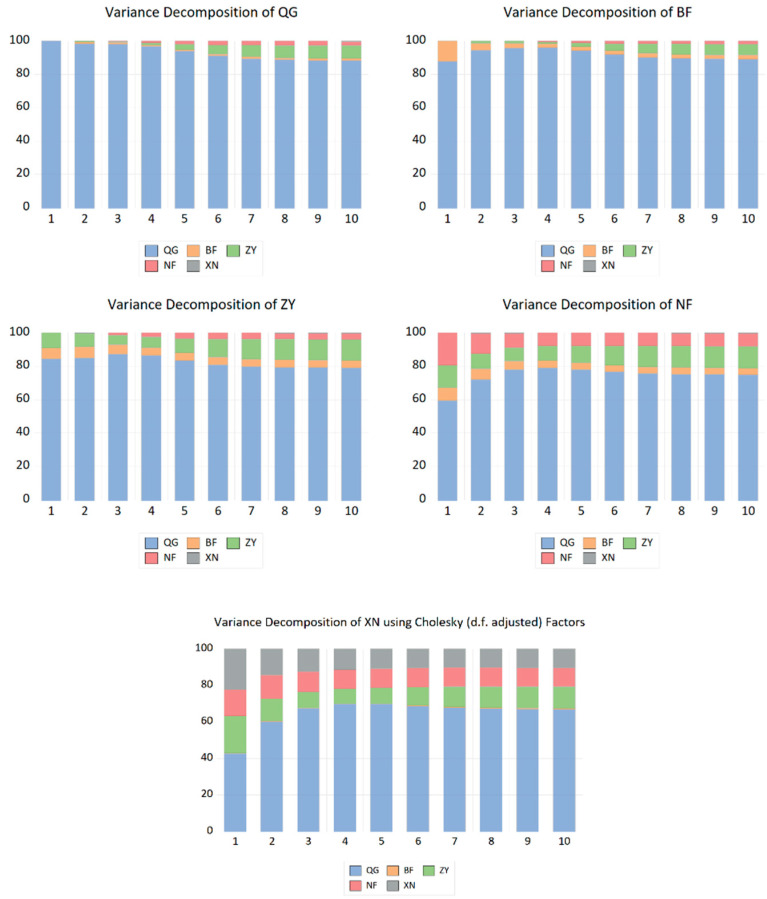
Changes in the Contribution Rate of Price Variance for Potatoes Nationally and Across Regions. Notes: QG represents the National, BF represents the Northern, ZY represents the Central Plains, NF represents the Southern, and XN represents the Southwest.

A variance decomposition analysis was conducted to assess the degree of price interaction between regions, indicating the changes in variance contribution rates across regions over a 10-period (month) lag (Figure 11). The Central China Second Crop Region exerted a significantly greater influence on national potato wholesale market price fluctuations than other regions, with its variance contribution rate stabilising above 5% from the fifth period onwards. The Southern China Second Crop Region contributed secondarily to national price changes, while the Southwest Mixed Cropping Region had the smallest impact. Beyond being substantially influenced by overall national price fluctuations, potato prices in each region exhibit distinct patterns: the Northern Single-Crop Region is most affected by price shocks originating from the Central Double-Crop Region, with this impact increasing over periods; the Southern Double-Crop Region exerts the second-greatest influence on its prices, while the Southwest Mixed-Crop Region exerts the least; The Central China second-crop region is significantly impacted by potato price shocks originating from both the Northern first-crop region and the Southern second-crop region. The Southern second-crop region experiences substantial price shocks from both the Northern first-crop region and the Central China second-crop region, with the influence from the latter showing an increasing trend. The Southwest mixed-crop region faces considerable price shocks from both the Southern second-crop region and the Central China second-crop region, similarly exhibiting an increasing trend in the impact from the latter.

In summary, potato prices across various regions are significantly influenced by national price fluctuations while also exhibiting varying degrees of mutual impact between regions. This indicates a degree of interdependence and interaction in potato pricing across different areas. The Central Plains Second Cropping Zone holds a significant position in the variance contribution rates of multiple regions, likely reflecting its pivotal role in potato distribution and consumption. Among all regions, the Southwest Mixed Cropping Zone exerts the least influence on variance contribution rates, potentially linked to the distinctive characteristics of potato production and market scale within this area.

## 5. Conclusions and Policy Implications

This study employs an Ensemble Empirical Mode Decomposition (EEMD) model, Spatial autocorrelation (ESDA) and Vector Autoregression (VAR) model to investigate spatial variations in wholesale market price fluctuations for potatoes across different regions of China from January 2014 to December 2024, alongside regional transmission mechanisms. The following key conclusions are drawn:

Firstly, China’s potato market exhibits pronounced regional price characteristics alongside discernible cyclical patterns. Across the entire 2014–2024 period, the Northern Single-Crop Region recorded the most intense short-term high-frequency fluctuations in wholesale potato prices, while the Southwestern Mixed-Crop Region indicated relative price stability. The Northern Single-Crop Region, Central China Double-Crop Region, Southern Double-Crop Region, and Southwest Mixed-Crop Region exhibited monthly wholesale price cycles of 9.43 months, 8 months, 5.74 months, and 5.5 months respectively for short-term fluctuations, alongside long-term cycles of 29.33 months, 29.33 months, 33 months, and 26.4 months. Analysis of variance contributions from high- and low-frequency components indicates that short-term, high-frequency fluctuations dominate potato wholesale market prices in the Central Plains second-crop region, while long-term, low-frequency fluctuations prevail in the Northern first-crop region, Southwest mixed-crop region, and Southern second-crop region.

Secondly, spatial heterogeneity in potato prices across China is pronounced, with national and regional potato prices primarily influenced by negative effects from other regions. Spatial positive correlations in China’s potato prices are relatively strong, exhibiting a distinct pattern of high-value concentration. Provinces showing positive correlations maintain relatively stable distribution, predominantly clustered within the East China and South China economic zones. Regarding regional transmission, national prices experience the most significant impact from internal shocks, which gradually diminish over time. Prices across all regions are significantly influenced by national price shocks. While initial impacts from other regions vary directionally, they generally shift to negative or diminish over time. Overall, regional potato price movements are first affected by synchronous changes in national prices and either their own or those of the Central Plains second-crop region while simultaneously experiencing countervailing effects from other areas.

Thirdly, the degree of interactive influence among regional potato prices exhibits considerable variation. The Central Region’s second-crop zone contributes far more significantly to national price fluctuations than other regions, followed by the Southern Region’s second-crop zone, with the Southwest Mixed-Crop Zone exerting the least influence. National and Central Region second-crop zone prices exert greater influence on Northern Region first-crop zone potato prices, followed by the Southern Region second-crop zone, with the Southwest Mixed-Crop Zone having the least impact. The national market, Northern First-Crop Region, and Southern Second-Crop Region exert substantial influence on potato prices in the Central Second-Crop Region. Similarly, the national market, Northern First-Crop Region, and Central Second-Crop Region significantly impact Southern Second-Crop Region prices. The Southwest Mixed-Crop Region is most affected by the national market, Southern Second-Crop Region, and Central Second-Crop Region.

Based on the research findings, the following recommendations are proposed:

Firstly, establish an intelligent monitoring and early warning system based on fluctuation cycles. This system should not only track absolute prices but also set differentiated dynamic alert thresholds—such as price volatility rates, month-on-month and year-on-year change rates—tailored to the identified short and long cycles across different regions. For instance, more sensitive short-term, high-frequency fluctuation alerts should be configured for the Northern First Crop Zone and Central Plains Second Crop Zone, while the Southwest and Southern Second Crop Zones should focus on deviations from long-term trends. Leveraging big data and artificial intelligence, integrate price data with multifaceted regional metrics including cultivated acreage, meteorological records, and warehousing logistics to distinguish normal cyclical fluctuations from abnormal disruptions caused by natural disasters or supply chain interruptions. Activate tiered responses according to alert levels: during blue alerts, issue market risk advisories to producers via information platforms; during yellow alerts, cooperatives and other entities are advised to adjust shipment schedules; for orange alerts and above, micro-regulatory measures such as inter-regional warehouse adjustments or temporary government stockpiling may be implemented as appropriate.

Second, implement precise inter-regional transmission pathway management. When prices are negatively impacted by other regions, this indicates potential non-benign competition or substitution shocks between areas. Policy focus should concentrate on major distribution hubs and wholesale markets. Strengthen brand and traceability development. In primary production areas such as the Northern First Crop Zone and Central Plains Second Crop Zone, vigorously promote the use of geographical indications and regional public brands, supported by stringent quality grading standards, establishing premium pricing for superior quality as market consensus. Market regulators should collaborate with industry associations to conduct specialised rectification campaigns and publicise actions against counterfeiting renowned regional brands or passing off inferior products as superior. Develop contract farming and medium-to-long-term agreements, encouraging large wholesalers and processors in consumption regions to establish stable supply relationships with primary production areas. Contractually locking in portions of output and prices can effectively mitigate sharp fluctuations and negative spillovers caused by short-term supply-demand mismatches.

Third, shape a regional industrial landscape characterised by functional complementarity and coordinated development. Define the strategic positioning of each region and implement differentiated support measures. Recognise and reinforce the Central Plains secondary crop region as the ‘price anchor’ and ‘market barometer’ for the national market, enhancing its price discovery function and market transparency to make fluctuations more predictable and thereby stabilise national market expectations. Prioritise support for the Northern primary crop region to develop modern storage and preservation facilities, extending the sales period and avoiding price collapses caused by concentrated market releases. Addressing the Southern Secondary Production Zone and Southwestern Mixed Production Zone’s characteristic long-term low-frequency fluctuations, guide farmers towards ‘time-differential’ and ‘variety-differential’ production. Leverage their climatic advantages to develop off-season and specialty varieties, filling market gaps to achieve differentiated competition. Establish regional coordination mechanisms to synchronise production schedules across zones during potential national supply-demand imbalances, fostering an integrated national industrial development framework.

This study indicates that potato price fluctuations in China exhibit pronounced spatial variability. This finding aligns with previous scholarly research on potatoes and other crops [49,51], confirming that prices for the same commodity are not uniform across different regions within a country, but rather exhibit spatial price differences. Examining long-term and short-term fluctuations separately indicates that the dominance of regional potato price movements over overall regional price fluctuations varies across different areas. Concurrently, the study observes that, similar to other agricultural products, potato prices exhibit certain spatial transmission patterns [63,67]. This study integrates the spatial differentiation of long-term and short-term potato price fluctuations with their transmission mechanisms within a unified analytical framework, thereby making a marginal contribution to systematically understanding the spatio-temporal regularities of agricultural commodity price fluctuations. Due to limitations in data availability and research length, this paper only explores the spatial differences and transmission mechanisms of potato prices in China from 2014 to 2024, without considering the influence of external factors such as population size and consumption habits. In light of this, the author plans to continue collecting fixed-point data on potato prices across different regions in China, and in future research, will further incorporate the influence of external factors such as population size and consumption habits, to further deepen the research on the spatial differences and transmission patterns of potato price fluctuations.

## Figures and Tables

**Table 1:** Regional Characteristics and Provincial Distribution of Potato Production in China.

Region	Production Characteristics	Province
Northern Single-Crop Zone (BF)	Cultivated only once per year, this summer crop is sown in spring and harvested in autumn. Sowing generally occurs between April and early May, with harvesting taking place from September to early October.	Heilongjiang, Jilin, Hebei, Shanxi, Inner Mongolia, Ningxia, Gansu, Shaanxi, Qinghai, Xinjiang, Beijing
Central Plains Double-Crop Zone (ZY)	The district practises spring and autumn cultivation. Spring production involves sowing from late February to early March, with harvesting occurring from May to mid-June; autumn production entails sowing in August, followed by harvesting in November.	Liaoning, Henan, Shandong, Jiangsu, Zhejiang, Anhui, Jiangxi, Tianjin, Shanghai
Southern Double-Crop Zone (NF)	Make greater use of the fallow winter period following rice harvest for potato cultivation, implementing autumn or winter sowing. For autumn sowing, plant in late October and harvest from late December to early January; for winter sowing, plant in mid-January and harvest in mid-to-late April.	Guangdong, Guangxi, Hainan, Fujian
Southwestern Mixed-Crop Zone (XN)	In high-altitude mountainous regions, cultivation typically follows a single-season pattern of spring sowing and autumn harvesting; whereas in low mountains, river valleys or basins, a two-season cultivation system is more common. Given the complex terrain and pronounced vertical climatic variation, potato cultivation in these areas comprises either a single-crop or a double-crop system.	Yunnan, Guizhou, Sichuan, Tibet, Hunan, Hubei, Chongqing

**Table 4:** ADF Unit Root Test.

Sequences	t-Statistic	Critical Values at Different Levels of Significance			(C, T, K)	Stability Testing
		1%	5%	10%		
National	−5.686	−4.038	−3.448	−3.149	CT3	Steady
Northern	−6.024	−4.039	−3.449	−3.150	CT3	Steady
Central Plains	−6.177	−4.038	−3.448	−3.149	CT3	Steady
Southern	−5.004	−4.038	−3.448	−3.149	CT3	Steady
Southwest	−3.578	−4.037	−3.448	−3.149	CT3	Steady

Notes: (C, T, K) denotes the test model, where C represents the intercept term, T denotes the trend term, K indicates the lag term, and 0 signifies the absence of either intercept or trend.

**Table 5:** Model Lag Order.

Lag	LR	FPE	AIC
0	NA	1.19 × 10^−9^	−6.358048
1	421.4771	4.10 × 10^−11^	−9.727789
2	61.36510 *	3.53 × 10^−11^ *	−9.879354 *
3	33.30259	3.92 × 10^−11^	−9.781732

Note: ‘*’ denotes the optimal lag order.

## Data Availability

The data presented in this study are available upon request from the corresponding authors.
